# A Wireless Flexible Sensorized Insole for Gait Analysis

**DOI:** 10.3390/s140101073

**Published:** 2014-01-09

**Authors:** Simona Crea, Marco Donati, Stefano Marco Maria De Rossi, Calogero Maria Oddo, Nicola Vitiello

**Affiliations:** The BioRobotics Institute, Scuola Superiore Sant'Anna, viale Rinaldo Piaggio 34, Pontedera (PI) 56025, Italy; E-Mails: m.donati@sssup.it (M.D.); s.derossi@sssup.it (S.M.M.D.R.); oddoc@sssup.it (C.M.O.); n.vitiello@sssup.it (N.V.)

**Keywords:** sensorized insole, plantar pressure distribution, gait analysis, real-time gait monitoring, wearable sensor

## Abstract

This paper introduces the design and development of a novel pressure-sensitive foot insole for real-time monitoring of plantar pressure distribution during walking. The device consists of a flexible insole with 64 pressure-sensitive elements and an integrated electronic board for high-frequency data acquisition, pre-filtering, and wireless transmission to a remote data computing/storing unit. The pressure-sensitive technology is based on an optoelectronic technology developed at Scuola Superiore Sant'Anna. The insole is a low-cost and low-power battery-powered device. The design and development of the device is presented along with its experimental characterization and validation with healthy subjects performing a task of walking at different speeds, and benchmarked against an instrumented force platform.

## 1. Introduction

Gait analysis is the systematic study of human walking, performed by collecting kinematic and kinetic data that describe and characterize it. Gait analysis is applied in different fields, such as in the clinical environment, where it is fundamental for the assessment of gait pathologies [1–3], the prevention of pressure ulcers in diabetes [4,5] or the assessment of the course of an orthopaedic disease. In addition, gait analysis carried out for sport purposes is aimed at helping athletes to gain a high level of performance [6–8], while minimising the risk of painful injuries to shins and joints [9]. Finally, scientific research laboratories use gait analysis with the aim to study mechanisms of human musculoskeletal system and cerebral apparatus. Each of these application fields uses different gait analysis techniques to pursue specific aims.

In the state of the art, three main solutions for plantar pressure monitoring are proposed: force platforms, pedobarographs and pressure-sensitive foot insoles [7]. Force and pressure platforms are very reliable and accurate devices, thanks to their very sensitive and high-frequency sensors (sensitivity is up to 1 μN, sampling frequency can reach 200 Hz); these devices can be used for both static and dynamic studies, like for assessing balance, posture and gait. Pedobarographs are characterized by extremely high spatial resolution, that can reach 1 mm [8]. Nevertheless both force platforms and pedobarographs are affected by several limitations such as high encumbrance, high weight and the lack of portability [10], which restrict their application to clinical or research laboratories. Moreover, force platforms are affected by the "targeting" effect, that significantly alters the normal gait of the subjects [11].

When a high portability is desired, or measurement of pressures at foot-shoe interface is required, pressure-sensitive insoles appear to offer the best trade-off in order to perform gait analysis. Their use is however limited to applications that do not need extremely precise measurements. Two main aspects are important when dealing with pressure-sensitive insoles: (1) the technology used for sensors; (2) the actual information that can be extracted.

In the last years, examples of sensorized insoles based on different sensing technologies have been developed and commercialized [12,13]: F-Scan^®^ system (Tekscan^®^, South Boston, MA, USA) uses force-sensing resistors (FSRs) [14]; the ParoTec^®^ system (Paromed^®^, Neubeuern, Germany) utilizes piezoresistive sensors [15]; the Pedar^®^ system (Novel^®^ GmbH, Munich, Germany) uses embedded capacitive sensors [16]. Despite that fact all of these systems have shown their usability in gait analysis applications, some limitations were pointed out, such as: (i) the flexible contact surface may distort unpredictably, causing undesired variations of the sensor response; (ii) the output may drift when the load is applied for long time, mainly due to the heat inside the shoe; and (iii) subject-specific calibration procedures may be needed and may alter measurement accuracy [17–20]. The scientific interest in the biomechanical evaluation of gait using portable devices is also evident when considering that many research laboratories are currently trying to develop their versions of insoles, with different technologies and different requirements [21–23]. The limitations of these research prototypes are mostly three: first, they usually have a relatively small number of sensitive elements, which are positioned in correspondence of specific anatomical *reperi*, and lead to a reduced spatial resolution and a consequent difficulty to reconstruct an accurate pressure map under the foot sole. Second, these prototypes require time-consuming subject-specific calibration procedures. Finally, in some cases, the devices store acquired data into an internal memory without an on-line data transfer to a remote computing/storing unit thus preventing them from being used in applications of real-time gait analysis [24,25].

One of the ultimate goals of gait analysis though pressure-sensitive insoles is the detection of gait events, e.g., heel strike, mid-stance, toe-off [26,27]. These events are important in order to extract biomechanical features for clinical diagnosis (e.g., gait speed, temporal duration of stance/swing, gait symmetry) and their variability over gait cycles, as well as walking conditions (e.g., speed, cadence) and locomotion tasks (e.g., ascending/descending stairs, sit-to-stand, stand-to-sit) [28]. However, sensorized foot insoles can also be used in other applications such as activity recognition (e.g., in ambient assisted living) [29], real-time control of robotic systems (e.g., in lower-limb powered prosthetic/orthotic devices), or the setup of rehabilitation strategies (e.g., using functional electrical stimulation) [30].

In this study we introduce the design and development, the experimental characterization and the benchmarking against an instrumented force platform of a novel flexible in-shoe device for real-time monitoring of plantar pressure distributions. The sensor technology relies on an optoelectronic transduction principle: a light emitter faces a photodiode as light receiver, they are covered by a shell made of opaque silicone which deforms under the effect of an external force occluding the light path. An extended abstract of this work was previously presented in a conference paper [31], where we gave a concise overview of the system design and architecture. Furthermore, in a more recent journal paper [32], we reviewed the opto-electronic pressure-sensitive technology and reported about the pressure-sensitive insole as a case-study application, by briefly recapping the work presented in [31]. Finally, the proposed pressure-sensitive insole was also experimented to validate methods of gait segmentation [33,34], techniques of sensory fusion for decoding motion intentions in healthy subjects [35], and to develop an augmenting feedback system for lower-limb unilateral transfemoral amputees [36].

The proposed system advances existing devices by integrating the following features: first, the sensing technology is not sensitive to the temperature, thus there is no drift in the output over prolonged recording sessions. The sensing technology does not need amplifiers so that conditioning electronics are not heavy and can be located on the shoe, and the subject has no need to wear any instrumented belt. Second, the system does not need repeated calibrations during long-duration acquisitions: calibration is performed just once in the lifetime of the pressure-sensitive insole, and—as a consequence—the system is easy to use. Third, the system has an appropriate spatial resolution (1 cm^2^), data are sampled at a relatively high sampling frequency (100 Hz) and its lifetime is sufficient to allow prolonged recording sessions such as the ones carried out in [33–36].

The paper is organized as follows: Section 2 describes the design and development of the device. Section 3 describes the experimental validation of the pressure-sensitive insole. Section 4 presents the results, that are then discussed in Section 5. Finally, in Section 6 we draw the conclusions and offer a perspective on future uses and further development of the system.

## 2. The Pressure-Sensitive Foot Insole

### 2.1. System Functional Requirements

The design of the pressure-sensitive insole addressed three main functional requirements: first, the pressure distribution under the foot sole should be estimated with a relatively high spatial (1 cm^2^) and temporal (0.01 s) resolution; in particular, the sensing area should be large enough to allow an accurate estimate of the spatial coordinates of the center of pressure (*CoP*) and the vertical ground reaction force (*νGRF*), which are relevant variables to assess the gait biomechanics [37]. Second, it is desirable that the measurement system is a self-standing wearable wireless system; at this regard, we aimed at developing a measurement apparatus that could be entirely integrated in the shoe, and able to transmit all relevant data wirelessly to a remote data storing/computing unit. Finally, the system should be battery operated and ensure an autonomy of at least eight hours: this is indeed desirable to enable the use of the system for prolonged recording sessions (e.g., monitoring the gait in activities of daily living) and for feeding data to the control system of robotic prostheses/exoskeletons [38].

### 2.2. System Architecture

**Figure 1.**
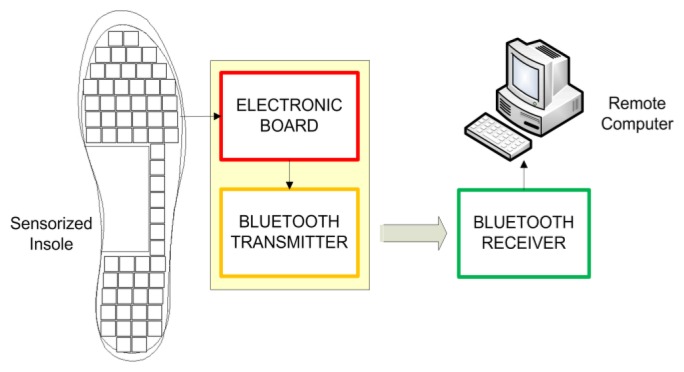
Overview of the system architecture.

The pressure-sensitive foot insole comprises two main parts: the *transduction unit* and the *on-board electronics* for signal conditioning and data transmission. A conceptual description of the system architecture is given in Figure 1.

The *transduction unit* consists of two main parts: (i) a black-dyed opaque silicone layer divided into 64 cells; and (ii) a 0.2-mm-thick printed circuit board (PCB) which houses the optoelectronic components. The sensing technology was developed at Scuola Superiore Sant'Anna over the last five years [32,39,40] for measuring the interaction pressure at the human-robot physical interface of the NEUROExos robotic exoskeleton for upper-limb rehabilitation [41–44] and the LOPES lower-limb active orthosis [45,46].

**Figure 2.**
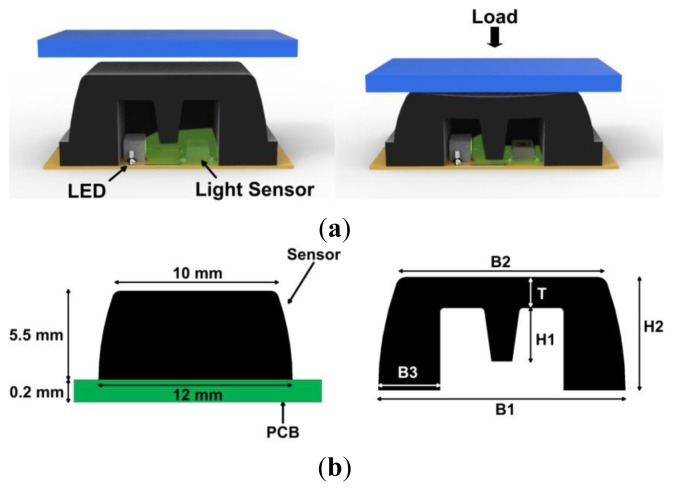
(**a**) Overview of the sensitive element and its functioning principle; (**b**) cross-section of the silicone cover; for the pressure-sensitive elements we chose the following values for the constructive parameters: B1 = 12 mm, B2 = 10 mm, B3 = 3 mm, H1 = 2.6 mm, H2 = 5.5 mm, T = 1.5 mm.

With reference to [32], we built the pressure-sensitive insole upon a modified version of the sensing element of the second generation of pressure-sensitive pads (PSP), namely PSP2.0. The transduction unit consists of independent silicone cells—the sensitive elements. The silicone cell has the shape of a pyramidal frustum with a square basis and an internal central curtain (Figure 2). Each cell covers a light emitter and a light receiver diodes, soldered on the PCB. The light emitter is a high-luminosity green LED (OSA Opto Light GmbH, Berlin, Germany [47]); the receiver is an ambient-light photodiode (Avago Technologies Ltd., San Jose, CA, USA [48]) and is equipped with an embedded temperature-compensation circuit which prevents the output signal from drifting over a wide operating range (10 °C–60 °C): this is suitable for all indoor applications and majority of outdoor scenarios.

The transduction mechanism acts as described in the following: when a load is applied on the top surface of the cover, the silicone bulk deforms itself and the curtain gradually closes the light path between the emitter and the receiver, and thus the output voltage changes. The sensor thus works as a force-to-voltage transducer. The dimension of the frustum base is 12 × 12 mm^2^, while the top face is 10 × 10 mm^2^, and the height is 5.5 mm (Figure 2b). The contact surface provides a spatial resolution of 1 cm^2^.

In the current prototype, differently from the PSP2.0 described in [32], in order to reduce the sensitivity to the tangential loads (which arise during walking mostly as a consequence of the push-off and can affect the sensor output) we addressed the following three changes in the shape and structure of the silicone bulk of the sensitive element: (i) we added a new geometrical parameter, *i.e.*, the thickness of the frustum base (B3 in Figure 2b); (ii) we changed the values of the other parameters; (iii) we employed a stiffer silicone rubber (Sorta Clear 40, Shore 40 A, Smooth-On Inc., Easton, PA, USA). Therefore the shape of the cover is identified by six geometrical parameters: (i) the side of the lower base B1; (ii) the side of the upper face B2; (iii) the thickness of the base B3; (iv) the thickness of the upper face T; (v) the height of the curtain H1; (vi) and the height of the frustum H2. By changing the mentioned geometrical parameters and/or modifying the mechanical properties of the silicone, the sensitivity of the sensor to the applied load as well as the measurable range of forces change. For the sensorized insole, we assumed a working range for each sensor of 0–500 kPa [5]. We identified the values of the geometrical parameters by means of iterative simulations using a 3D finite-element model, as explained in [32].

**Figure 3.**
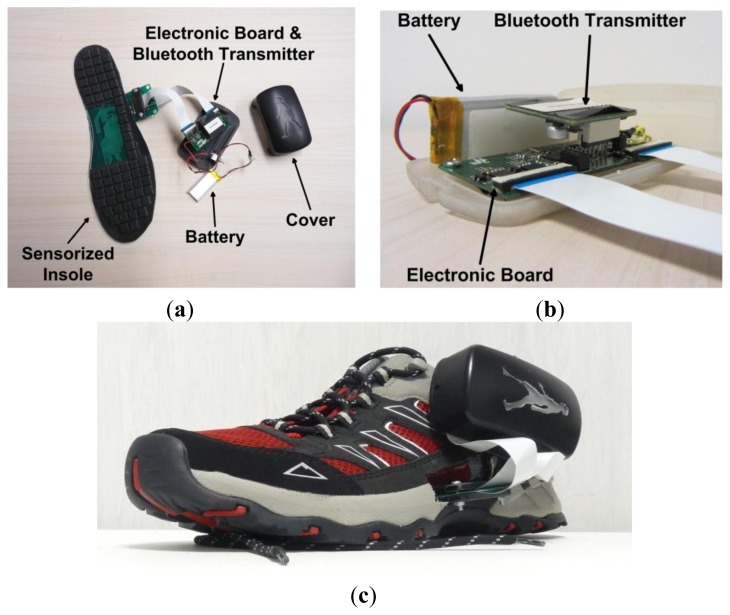
(**a**) Overview of the device: sensorized insole connected to the electronic board through flat cables, Bluetooth transmitter, Li-Ion battery; (**b**) detail of the electronic board and Bluetooth transmitter connected together and placed into a box; (**c**) overview of the device set up into the shoe.

An overview of the *electronic board*, purposively engineered by Robotech (Peccioli, Italy) for the pressure-sensitive insole, is shown in Figure 3. The main components of the board are: (i) four analog-digital converters (ADC) to perform high-frequency sampling and digitalization of the signals; (ii) a STM32F103x8 microcontroller that performs all the computation; (iii) a power socket to power the board either with external 3.6 V power supply, or with an external Lithium-Ion battery; (iv) a communication socket to connect the acquisition board with the communication board through a serial UART protocol.

The electronic board performs the following operations:

(1) sampling of the 64 analog signals at 1.2 kHz frequency through the four 16-channel ADCs;
(2) low-pass filtering (cut-off: 40 Hz) and down-sampling to 100 Hz;
(3) voltage-to-force conversion of the output signal from each sensitive element based on the characterization curve reported in the next sub-section;
(4) calculation of the total *νGRF* and coordinates of the *CoP*;
(5) data transmission by means of a Bluetooth connection to remote receivers at 100 Hz.

For the calculation of the *νGRF* and *CoP* coordinates, first, the output voltage *ν_i_* of the i-th sensitive element was de-offset and converted into a force *F_i_*:

(1) $$\{\begin{array}{c} F_{i}=0\;N,\;\text{if}\;v_{i}>-0.02\;\text{V}\\ F_{i}=21.386\;N\cdot e^{4.834\cdot \left(v_{i}/V\right)}-22.30\;N\cdot e^{-0.401\cdot \left(v_{i}/V\right)}\;,\;if\;v_{i}\leq -0.02\;V \end{array}$$

then *νGRF* and *CoP* coordinates were calculated as follows:

(2) $$\{\begin{array}{c} \nu \text{GRF}=\sum_{i=1}^{64}F_{i}\\ CoP_{x}=\sum_{i=1}^{64}F_{i}\cdot x_{i}/\nu \text{GRF}\\ CoP_{y}=\sum_{i=1}^{64}F_{i}\cdot y_{i}/\nu \text{GRF} \end{array}$$

with *x_i_* and *y_i_* being the spatial coordinates of each sensitive element. Notably, *CoP_x_* and *CoP_y_* (*x* and *y* coordinates of the *CoP*) were set to “Not a Number” (NaN) when *νGRF* >−20 N (we assumed *νGRF* = 0 N if *νGRF* >−20 N) Furthermore, we assumed that *x* and *y* axes identify respectively the medial-lateral and antero-posterior foot sole directions; in particular the *y* coordinate spans from 0 mm, when the *CoP* is under the toe, to 250 mm, when the *CoP* is under the heel.

The current absorption of each insole (sensors and electronics) is about 150 mA at 3.6 V (nominal power is about 0.54 W): a battery (size: 25 × 50 × 3 mm) with a capacity of 2,000 mAh (*i.e.*, a low-cost cell-phone battery) ensures an autonomy of about 20 h. The array of sensitive elements is connected to the electronic board through two flat cables (each with 32 analog channels) carrying unamplified analog voltage signals.

### 2.3. Experimental Characterization of the Sensitive Element

Given the different shape of the silicone rubber bulk, we carried out a novel experimental characterization of the sensitive elements, aimed at assessing the force-to-deformation behavior of the silicone cover, as well as at constructing the force- (or pressure-) to-output voltage curve of each sensor. The force-to-output voltage characterization was performed by using a 3-axial platform (TAP) machine, developed at The BioRobotics Institute of Scuola Superiore Sant'Anna (Pisa, Italy), equipped with a mono-axial load cell (LCM300, Futek, Irvine, CA, USA), and a rigid flat indenter. While applying a pre-defined set of deformations on the sensitive element, we recorded the reaction force and the output voltage of each sensor.

Since the silicone cover of the sensorized insole was obtained by casting silicone into a single mold, we expected that the force-to-output voltage behaviour of all sensitive elements could differ among them within a narrow range. As a consequence, we could identify an aggregate calibration function (*i.e.*, the force-to-output voltage curve) by averaging the behaviour of all sensitive elements.

**Figure 4.**
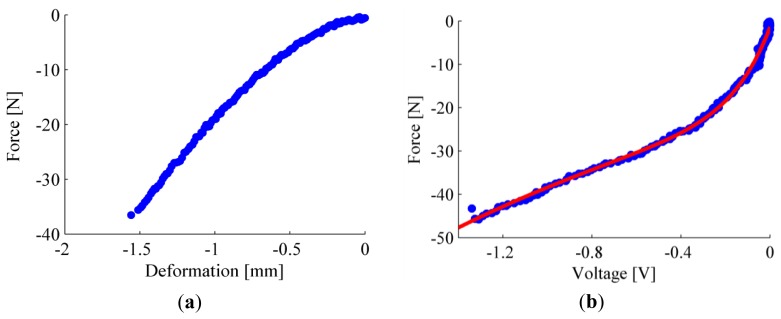
Characterization of one representative sensitive element: (**a**) quasi-static force-to-deformation characterization; (**b**) quasi-static force-to-output voltage curve, experimental data of one selected sensor (blue dots) and fitting model (solid red line).

In order to identify the quasi-static force-to-output voltage curve—for each sensitive element—we applied a deformation in the range 0–1.5 mm, with a loading speed set to 0.084 mm/s (*i.e.*, ∼5 mm/min). Resulting data from each sensitive element were fitted by the sum of two exponential functions (*i.e.*, *F* = *A*_1_*e*^*c*_1_*ν*^+ *A*_2_*e*^*c*_2_*ν*^, where *F* is the applied force and *ν* is the output voltage), which was found to be the best compromise in terms of complexity and goodness of fit (Matlab^®^ *cftool*). Figure 4 reports the experimental curves for one representative sensitive element, along with its numerical model. The average value of the coefficients of the numerical model of all 64 sensitive elements (along with average values of the parameters showing the goodness of the fit, *i.e.*, RMSE and R^2^) and the parameters of the aggregate calibration model are summarized in Table 1.

### 2.4. Data Recording, Graphical User Interface and Gait Segmentation Algorithm

**Figure 5.**
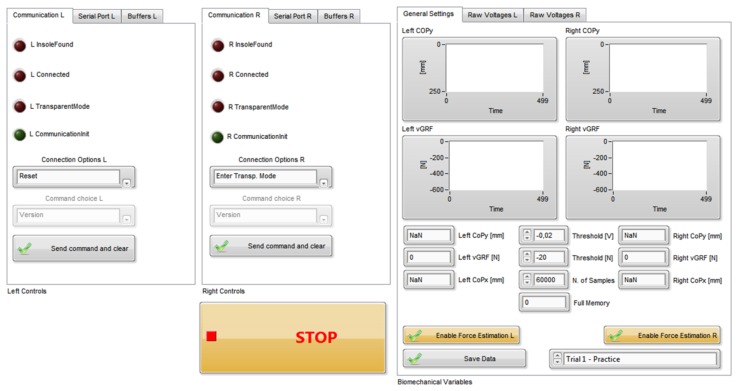
Graphical user interface developed in Labview environment.

Data from the pressure-sensitive insoles are received, real-time processed and stored on the remote PC by means of a custom Labview routine (National Instruments Inc., Austin, TX, USA) with a graphical user interface (GUI). Data from the pressure-sensitive insoles can be received by any remote device (PC, tablet or smartphone) equipped with custom Bluetooth receivers, engineered by Robotech. A 921.6 Kbit/s connection is required to sustain a 100 Hz communication rate. An overview of the GUI is given in Figure 5.

Through the developed GUI the experimenter can send commands to the on-board microcontroller of the device to initiate (or stop) the data acquisition, to de-offset raw voltages, and information on the status of the battery.

The GUI also allows the user to: (i) real-time display the foot pressure map; (ii) show the graph of the *νGRF*, and of the instantaneous position of the *CoP*; (iii) display the force applied on each singular sensitive element. Finally, the custom Labview routine is also deputed to execute a real-time gait segmentation: collected biomechanical variables (*i.e.*, *νGRF* and *CoP_y_*) are used to identify three gait phases (namely “*Stance 1*”, “*Stance 2*” and “*Swing*”), in accordance to a simplified formulation of the model proposed by Perry and Davids [49]:

(1) Stance 1 (ST1) starts with the heel-strike and ends when the body weight is aligned with the forefoot; with reference to the Perry and Davids model, ST1 groups the phases *initial contact* and *mid-stance*;
(2) Stance 2 (ST2) starts from the end of ST1 and ends with the toe-off; with reference to the Perry and Davids model, ST2 groups the phases *terminal stance* and *pre-swing*;
(3) Swing (SW) starts with toe-off and ends with heel-strike; SW coincides with the *swing* phase of the Perry and Davids model.

The gait-segmentation algorithm is addressed by means of the following set of Equations:

(3) $$\{\begin{array}{c} CoP_{x}=CoP_{y}=\text{NaN}\to \text{phase}:\;\text{SW}\\ \nu \text{GRF}\;\leq -20\;\text{N}\;\text{and}\;CoP_{y}>125\;\text{mm}\to \text{phase}:\text{ST}1\\ \nu \text{GRF}\;\leq -20\;\text{N}\;\text{and}\;CoP_{y}\;\leq 125\;\text{mm}\to \text{phase}:\text{ST}2 \end{array}$$

The empirical threshold to differentiate between stance and swing was set to −20 N after preliminary experiments. This threshold allows one to detect the heel strike and toe off events with a few milliseconds of delay (about 30 ms), and prevents recognition of false positives due to the noise of the sensors.

## 3. Experimental Validation

### 3.1. Experimental Protocol

Two healthy subjects volunteered to take part to the experimental validation of the sensorized insole. Table 2 summarizes the main features of the two subjects. Both the subjects had no gait impairment and signed an informed consent. Upon arrival subjects wore comfortable sportswear and athletic shoes, equipped with pressure-sensitive foot insoles. They were asked to walk for some minutes to become familiarized with the equipment.

Each subject was asked to walk on a straight line, starting from a still position at one end of the room, and ending at the opposite end (the path was about 10 m long). In particular, subjects were requested to repeat the ground-level walking task for 15 times at a self-selected slow speed, and for 15 times at a self-selected normal speed.

The walkway was also equipped with a force platform in order to perform a mid-gait protocol and compare insole measurement with the output of a commercial force plate (AMTI, Watertown, MA, USA), which is considered as the standard reference in the field of kinetic measurements for gait analysis. Subjects were specifically instructed to walk without taking care of hitting the force plate, in order to avoid the problem of “targeting” [11].

Raw voltages and biomechanical variables (namely the coordinates of the *CoP* and the *νGRF*) from both pressure-sensitive insoles, along with online computed gait phases, were synchronized with force plate output data through a synchronizing event recorded by all apparatuses. All data were then stored in a file for offline analysis.

### 3.2. Data Analysis

Recorded data were analyzed as follows. First, for each insole, from all 64 force values we reconstructed a time-changing qualitative map of the pressure distribution under the foot sole. In particular, pressure maps were created by applying a mesh grid to the raw map of forces (through a custom Matlab^®^ routine), in order to 3-dimensionally connect all the collected samples. No smoothing techniques were applied neither to regularize the surface nor to remove outliers.

**Figure 6.**
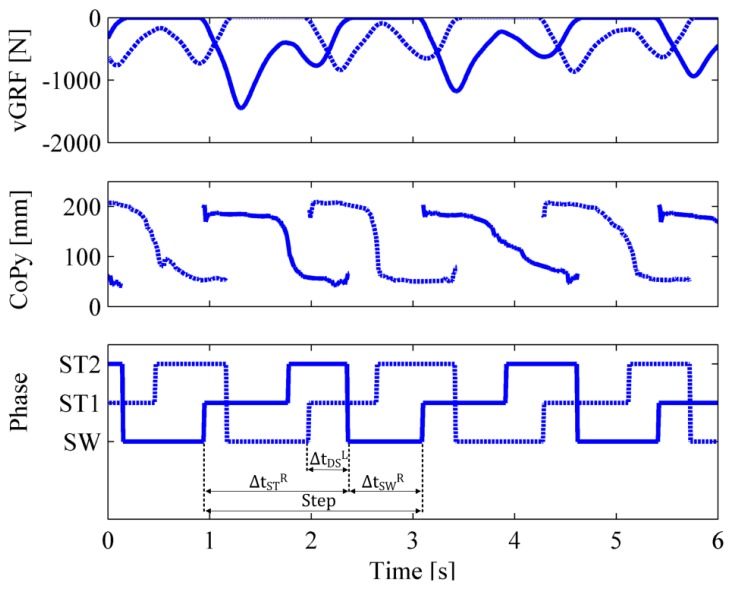
Extraction of temporal gait parameters. The top and mid panel depict the gait parameters (*νGRF* and *CoP_y_* respectively) acquired from the right (solid blue line) and left (dotted blue line) pressure-sensitive insole. The bottom panel shows the results of the online classification in gait phases and the use of these phases to calculate temporal gait parameters for the right ( $\Delta t_{ST}^{R}$, $\Delta t_{SW}^{R}$, $\Delta t_{DS}^{L}$) and left ( $\Delta t_{ST}^{L}$, $\Delta t_{SW}^{L}$, $\Delta t_{DS}^{R}$) foot.

Second, by combining the online computed gait phases of both pressure-sensitive insoles, we calculated the following relevant gait parameters: (i) stance and swing duration of both feet; (ii) duration of the double-support phases; and (iii) step cadence of both feet. For each trial, the first and the last two steps were removed from the analysis in order to process only data related with steady-state steps: for each foot, a step is identified as the time interval between two heel strikes. Figure 6 describes the extraction of temporal gait parameters, based on the online computed gait phases.

For each foot, the duration of the stance phase ( $\Delta t_{ST}^{L}$, for the left foot, and $\Delta t_{ST}^{R}$, for the right foot) was computed by summing up the duration of the phases “ST1” and “ST2”. The duration of the swing phase ( $\Delta t_{SW}^{L}$, for the left foot, and $\Delta t_{SW}^{R}$, for the right foot) was equal to the duration of the phase “SW”. The duration of the double-support phase preceding a left-foot single support $\left(\Delta t_{DS}^{L}\right)$ was computed as the time interval in which the left foot was in the phase “ST1” and the right foot was in “ST2”. At the same way, the duration of the double-support phase preceding a right-foot single support $\Delta t_{DS}^{R}$ was computed as the time interval in which the left foot was in the phase “ST2” and the right foot was in “ST1”. Right and left step cadence (*C_R_* and *C_L_*) were computed as $C_{L}=1/(\Delta t_{ST}^{L}+\Delta t_{SW}^{L})$ and $C_{R}=1/(\Delta t_{ST}^{R}+\Delta t_{SW}^{R})$

Finally, for all the steps that were fully recorded by the force platform, we compared the *νGRF* profile computed by the insole with the one measured by the force platform: data of all selected steps were re-sampled in 100 samples, and averaged across all steps.

We calculated different average *νGRF* profiles for slow and normal speeds, as well as for the two different subjects. The comparison with the data from the force plate was addressed by computing the normalized root mean square error (NRMSE) and the Pearson correlation (PC) coefficient. Being the root mean square error defined as $\text{RMSE}=\sqrt{\sum_{i=1}^{n}{\left(\text{vGR}F_{i}^{in}\;\text{vGR}F_{i}^{fp}\right)}^{2}/n}$, then we computed the NRMSE as follows: $\text{NRMSE}=\sqrt{\text{RMSE}/\left(\text{max}(\nu GRF^{in})-\text{min}(\nu GRF^{in})\right)}$, where *νGRF^in^* and *νGRF^fp^* denote the *νGRF* measured respectively by the insole and the force plate, and *n* is the number of observations. Furthermore, we calculated the mean absolute error (MAE) between the stance phase duration computed from the force-platform $\left(\Delta t_{ST}^{fp}\right)$ and the insole data $\left(\Delta t_{ST}^{in}\right)$, being the stance phase duration the only temporal gait parameter that we could compute from both insole and force platform data.

## 4. Results

### 4.1. Pressure Maps

**Figure 7.**
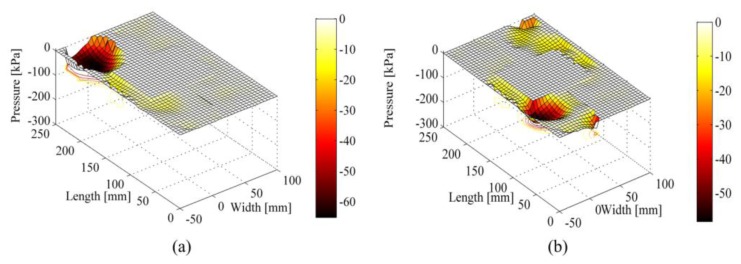
Pressure maps under the foot at different gait phases. (**a**) Weight-acceptance phase of the right foot. The weight is distributed on the heel region. The left foot is swinging; (**b**) Push-off phase of the right foot. The weight is distributed on the right forefoot. The left foot is starting to contact the ground.

An example of pressure maps that can be extracted from the developed pressure-sensitive insoles is reported in Figure 7: the reported maps depict typical under-sole pressure patterns for Subject #1 during the weight acceptance phase (Figure 7a—pressure is under the heel—and the push-off phase—pressure distribution is mostly under the forefoot area (Figure 7b).

### 4.2. Gait Parameters

Averaged values of computed gait parameters are summarized in Table 3. The mean and standard deviation of each parameter are reported for both slow and normal speeds.

Subject 1 walked with a step cadence of 0.80 Hz (i.e., 48 steps per min) during the slow trials, and slightly increased the cadence to 0.92 Hz (i.e., 56 steps per min) during the normal-speed trials. Coherently, the comparison of the results in the two conditions revealed a diminished stance and swing duration for both feet. On the other hand, stance and swing duration expressed in percentage of gait stride did not change significantly between the two conditions.

Results for Subject #2 were consistent, with slight differences. Indeed, as for Subject #1, from self-selected slow-speed to normal-speed trials the step cadence increased from about 0.76 Hz (i.e., 46 steps per min) to 1.04 Hz (i.e., 62 steps per min), with the duration of the stance and swing phases significantly decreasing. Differently from Subject #1, a higher cadence also resulted in a change of the gait pattern: the percentage of stance duration significantly increased, while the percentage of swing duration significantly decreased.

### 4.3. *νGRF* Profiles

**Figure 8.**
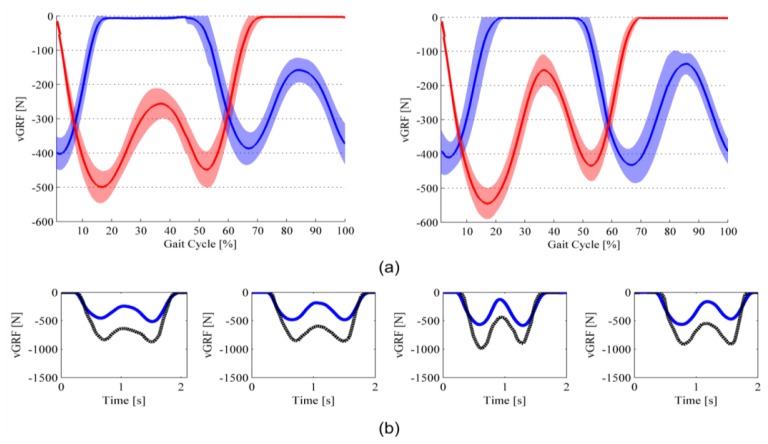
*νGRF* profiles of Subject #1. Red line is the left foot; blue line is the right foot. (**a**) Average curve during slow- (on the left panel) and normal- (on the right panel) speed ground-level walking; (**b**) Comparison between the *νGRF* profile measured through the sensorized insole (solid blue line) and the force platform (dotted black line) in four different steps.

**Figure 9.**
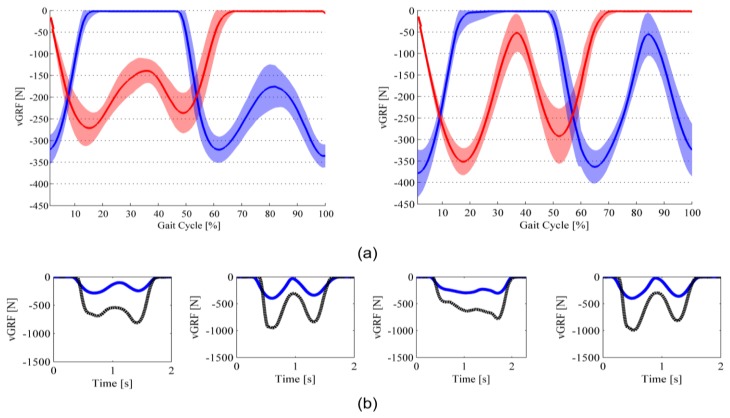
*νGRF* profiles of Subject #2. Red line is the left foot; blue line is the right foot. (**a**) Average curve during slow- (on the left panel) and normal- (on the right panel) speed ground-level walking; (**b**) Comparison between the *νGRF* profile measured through the sensorized insole (solid blue line) and the force platform (dotted black line) in four different steps.

The *νGRF* profiles of steady-state steps for Subject#1 and Subject #2 are shown in Figures 8 and 9. *νGRF* profiles of both left and right feet are shown in Figure 8a and Figure 9a (data are averaged across all recorded steps—about 50 steps for each subject and each speed condition—(solid line), and shown along with the standard deviation contour (shadowed).

For the two different speed conditions, and for both subjects, *νGRF* profiles exhibit the physiological double-peak behavior: the first peak is recorded in correspondence of the end of the weight-acceptance phase, which occurs between 15% and 25% of the total stance time; the second peak is recorded in correspondence of the push-off phase, and occurs between 70% and 80% of the total stance time [49]. Coherently with human physiological biomechanics, with the walking speed increasing, the weight-acceptance peak increases, and the minimum force between the two peaks decreases [50]. For sake of clarity, it is worth noting that we are making reference to the absolute value of the recorded *νGRF*.

Figure 8b and Figure 9b report data from four different steps that compare the *νGRF* computed through the insole output data with the one from the force platform. In all selected steps, there are two common trends to highlight. On the one hand, the force measured by the force platform is higher than the one measured by means of the sensorized insoles. On the other hand, despite the difference in the recorded values, the *νGRF* profiles have the same qualitative pattern. Both the trends are confirmed by the computed NRMSE and Pearson correlation coefficient reported in Table 4: indeed, on average the NRMSE is about 80 (relatively high discrepancy between the profiles in terms of absolute value) and the PC coefficient is higher than 0.8 (low discrepancy between the profiles in terms of qualitative pattern). Table 4 also reports that $\text{MAE}(\Delta t_{ST}^{fp}-\Delta t_{ST}^{in})$ is on average equal to 0.03 s.

## 5. Discussion

### 5.1. Wearability of the System

In all the tests that were carried out, overall the developed apparatus resulted to be effective to perform gait analysis. In particular, the two subjects could easily wear the sensorized shoes and successfully walk: none of them reported any discomfort from wearing the shoes equipped with the pressure-sensitive insoles and walking for long periods. Furthermore, the placement of the electronic box on the lateral side of each shoe enhanced comfort and prevented the subjects from wearing any additional belt.

### 5.2. Sensing Technology: Advantages and Limitations

There is an increasing attention to the use of pressure-sensitive foot insoles for gait analysis because of their inherent advantage of wearability and portability (compared to more traditional instrumentations such as pedobarographs and force platforms), that allow one to make recordings in scenarios of activities of daily living [21]. In this work, we presented a new pressure-sensitive insole to be used for the assessment of gait performance and/or for real-time gait segmentation purposes. The proposed system integrates particular technological solutions at the level of the transduction unit, that allow to solve some of the main problems affecting other existing devices.

One major limitation of existing prototypes is the sensitivity of the transduction unit to the increasing temperature and humidity inside the shoe while walking [18]. The sensor technology that we used offered the advantage of being inherently non-sensitive to humidity and temperature, thanks to the light receiver we used [48]. The consequence is that during prolonged recording sessions we did not experience any drift of the sensor output, and therefore there is no need for repeated calibration procedures or signals de-offset, which is instead a limitation for other state-of-the-art pressure-sensitive insoles, which are based either on capacitive or resistive sensors [51,52]. On the contrary, the voltage-to-force calibration of the developed pressure-sensitive insole is performed once in the life of the prototype: this feature enhances the overall system usability.

Another significant advantage that derives from the choice of an optoelectronic transduction principle is the fact that each sensitive element has a non-amplified output voltage range of about 1.3 V. Thanks to this feature, the conditioning electronics thus does not require amplifiers: as a consequence, the conditioning electronic board has a relatively small size and can be housed on the instrumented shoe.

Despite the above mentioned strong points of the proposed pressure-sensitive technology, there are two main limitations that are worth discussing. The first limitation comes from the fact that we used a unique calibration curve for all 64 sensitive elements. Indeed, although we experimentally calculated the voltage-to-force curve for each sensitive element, we used a unique numerical model for all sensors. This choice resulted from the need to reduce the complexity of the firmware running on the onboard processing unit and was favored by relatively low variability of the force-to-voltage across all sensitive elements. On the other hand, this choice can affect the accuracy in estimating the *νGRF*: although on the single sensitive element the adopted numerical voltage-to-force model can lead to a relatively small RMSE (about 5% of the full-scale range Table 1), by summing up the error on all sensitive elements in the worst scenario (all sensors are indented with a load magnitude of about 50 N), the estimation of the *νGRF* can be affected by an error which can increase up to 160 N.

The second limitation derives from the noise threshold of −0.02 V which is necessary to apply when we compute both the *νGRF* and the coordinates of the *CoP* (see Equation (3)). Indeed, this threshold is crucial to prevent from getting false recognitions of initiation and termination of the stance phases (ST1 and ST2). However, as a consequence of a high slope of the force-to-voltage curve for small values of *ν_i_*, setting the output force *F_i_* to 0 N for *ν_i_* > −0.02 V causes a relatively high error in the estimation of *F_i_*, *i.e.*, from 0 N up to |*F_i_*| = |21.386 N *e*^4.834·(−0.02/V)^ − 22.30 N *e*^−0.401·(−0.02/V)^| = 3.06 N. This means that in the worst case in which all sensors are loaded with a load slightly lower than 3 N (absolute value), the estimation of the *νGRF* can be affected by an error ranging from 0 to 195.8 N.

The above two mentioned limitations suggest two future developments for the proposed insole. First, it is desirable to introduce a smarter fitting, that would allow the use of a different voltage-to-force curve for each sensitive element. Second, in order to minimize the error deriving from the application of a threshold to *ν_i_*, it is desirable to optimize the shape of the silicone cover, as well as the size of the curtain occluding the light path in such a way that the voltage-to-force curve has a smaller |*dF_i_*/*dν_i_*| for values of *ν_i_* close to 0 V.

### 5.3. *νGRF* Profile: Comparison with a Force Plate

The first outcome that it is possible to observe when we look at the *νGRF* profiles is that they exhibit the typical, physiological patterns, *i.e.*, the double-peak curve, with the first peak occurring at the weight acceptance phase and the second at the push-off. Moreover, in agreement with the state of the art higher gait speed is accompanied by larger peak forces and lower valley [50,53].

It is worth noting that Figure 8a and Figure 9a also show a slight difference between left and right foot insole *νGRF* profiles (in Subject #1 left-foot force peaks are higher than the ones from right foot; in Subject #2 right-foot force peaks are higher than the ones from left foot). The reason of these slightly asymmetrical gait patterns can be found in two facts. First, both subjects could have a slight gait asymmetry. Second, the errors in the estimation of the *νGRF* can be different for the left and right insoles as a consequence of: (i) slightly different placement of the insoles in the shoes; (ii) the RMSE of the voltage-to-force curve can change for the two set of 64 sensors: insoles are assembled by means of a hand-made process, which can lead to a certain variability in the parameters of Equations (1) and (2).

Finally, when we look at the comparison between the insole and force-platform *νGRF* profiles, the remarkable finding is the fact that—despite a significant difference in terms of actual values measurements, which is the consequence of the errors in the estimation of *νGRF* (see the discussion in Section 5.2)—insole and force-platform patterns showed a high qualitative correlation: this is evident in all plots of Figures 8b and 9b, also in the one (*i.e.*, third plot from left of Figure 9b) in which both the force platform and the insole recorded a *νGRF* profile lacking the typical double-peak feature. This is much important when we want to use the developed insoles for purposes of gait segmentation and basic analysis of the gait pattern [54]. Indeed, in order to reliably address a gait segmentation—and consequently a basic gait analysis—an important fact is the possibility to discriminate between major gait phases (ST1, ST2 and SW), and use the detected events to extrapolate relevant gait parameters (see Section 5.4).

High qualitative correlation of the insole to force-plate *νGRF* patterns enhanced the use of the presented insoles in several applications, such as: to investigate algorithms for automatic gait segmentation [33,34], to control robotic orthoses/prostheses (in [35] insole output data were used to successfully detect user intention to “initiate” or “terminate” the gait, and to develop a system for providing amputees with real-time feedback on the gait phase transitions [36]).

### 5.4. Gait Segmentation and Temporal Gait Parameters

While the task of detecting gait phases through observation can be trivial, however tedious, for a practiced human observer, it has been a challenge to create autonomous algorithms to consistently extract the events from data. The presented threshold-based algorithm was designed to reliably detect gait-phase transitions and allow a real-time segmentation. Despite it is a simple algorithm, the tuning of the thresholds was not straightforward: a wrong choice of the thresholds for both *ν_i_* and *νGRF* could lead to mistakes such as: (i) *CoP_x,y_* = NaN during the stance phase; (ii) *CoP_x,y_* ≠ NaN during the swing phase; or (iii) higher error in the estimation of *νGRF* (see Section 5.3). Therefore, preliminary experiments and the validation with the two healthy subjects were necessary and functional to prove that the technology—despite its limitations—can be successfully used to real-time segment the gait, even with a simple threshold-based algorithm.

Furthermore, it is worth mentioning that the choice of implementing a simple threshold-based segmentation algorithm derived from the analysis that more complex, artificial-intelligence black-box algorithms—such as the ones we developed in [33,35]—despite a higher number of detected phases, rely on the use of a large data set for training a classifier. The construction of such a data base requires the tremendous effort of an expert that has to manually segment a large amount of data: this could be undoubtedly a limiting factor to the use of our pressure-sensitive insoles in tasks of gait analysis where it is not requested to discriminate among more than three phases for each foot, namely *swing*, *early stance* and *late stance*.

Results of the experimental validation also proved that the temporal gait parameters calculated starting from the online gait segmentation are coherent with the reference data for health young subjects: our pressure-sensitive insoles are therefore suitable tools to assess gait parameters.

The basic gait parameters most frequently used to characterize gait are velocity, step length, step frequency and stance/swing duration (reference data for gait analysis of healthy subjects are provided in [49,55]). Since gait velocity and step length are not evaluable trough pressure-sensitive insoles, the comparison with those reference data can be done only by considering the step frequency, as well as the duration of stance and swing phases. If we look at the step frequency, data from our subjects are the typical ones for healthy (age: 23, 25) young subjects walking at a self-selected slow or normal cadence [55]. A similar outcome can be found if we look at the stance and swing duration [49]: right and left stance phases range between the 63% and 66% of the gait cycle (while swing phase results in the range between 34% and 37%), and the double support phases range from 12% to 16% of the gait cycle.

Suitability of insoles in the estimation of gait parameters is finally proved by the relatively small $\text{MAE}(\Delta t_{ST}^{fp}-\Delta t_{ST}^{in})$, which is about 0.03 s (5% of the stance duration in the normal speed trials). This error is mostly a consequence of the application of a threshold to *νGRF* for addressing the gait segmentation (see Equation (3)). This result—on the one hand—demonstrated a high precision of the system in the calculation of the stance duration and—on the other hand—indirectly proved the reliability of all the other temporal parameters calculated through the insoles, being the computation of the latter variables dependent on the accurate identification of the heel strike and toe-off, *i.e.*, the initial and final event of the stance phase.

## 6. Conclusions

We have presented the design, development and the experimental characterization of a novel flexible in-shoe device for real-time monitoring of plantar pressure distributions. The proposed system addresses the advancement of existing devices, presenting a sensing technology which is not sensitive to the temperature thus it does not need repeated calibrations during long-duration acquisitions; it also does not need any amplification of electrical signals. The system has appropriate spatial resolution and relatively high sampling frequency (100 Hz). Although we described some limitations of the device, we reported a detailed description of the outputs that can be obtained. From a clinical point of view, on one hand, the analysis of pressure distributions under the foot sole can be evaluated by looking at the pressure maps in different gait phases; on the other hand, quantitative gait parameters can be calculated starting from the real-time segmentation.

Future works will aim at optimizing the sensitive technology to reduce the error in the estimation of the *νGRF*; particular attention will be paid to optimize the shape of the silicone cover as well as the size of the curtain occluding the light path. On the other hand, the developed insoles will be further tested by benchmarking the current prototype against existing commercial-available technologies (e.g., F-Scan by Tekscan) and controlling smart active lower-limb prostheses and orthoses.

## Figures and Tables

**Table 1.** Fitting model of the force-to-output voltage curve.

	**Fitting Model Coefficients (with 95% Confidence Interval)**				**Fit Goodness**		

	A1	c1	A2	c2	RMSE [N]	R^2^	RMSE [%] of f.s.r.
Averaged Value over 64 Numerical Models	19.366 ± 5.526	6.745 ± 3.704	−20.458 ± 5.569	−0.596 ± 0.214	1.071 ± 0.501	0.988 ± 0.011	2.142 ± 1.002
Aggregate Calibration Model	21.386	4.834	−22.300	−0.401	2.719	0.932	5.438

**Table 2.** Subjects characteristics.

	**Shoe Size [EU]**	**Weight [kg]**	**Height [cm]**
Subject #1	42	82	172
Subject #2	42.5	73	170

**Table 3.** Gait parameters: for all computed parameters we report the average value and the standard deviation (μ±σ). $\Delta t_{ST}^{L}$, $\Delta t_{ST}^{R}$, $\Delta t_{SW}^{L}$, $\Delta t_{SW}^{R}$, $\Delta t_{DS}^{L}$, $\Delta t_{DS}^{R}$(for the definition see Section 3.2) are expressed both in (s) and (%) of the gait stride. Right and left step cadence (*C_R_* and *C_L_*) are expressed in [Hz].

	$\Delta t_{ST}^{L}[\text{s}]$[s] ([%])	$\Delta t_{SW}^{L}[\text{s}]$ ([%])	$\Delta t_{DS}^{L}[\text{s}]$ ([%])	*C_L_* [Hz]	$\Delta t_{ST}^{R}[\text{s}]$ ([%])	$\Delta t_{SW}^{R}[\text{s}]$ ([%])	$\Delta t_{DS}^{R}[\text{s}]$ ([%])	*C_R_* [Hz]
Subject #1	0.80 ± 0.06	0.46 ± 0.04	0.17 ± 0.02	0.80 ±	0.82 ± 0.07	0.42 ± 0.04	0.19 ± 0.06	0.81 ±
Slow speed	(63 ± 2)	(37 ± 2)	(14 ± 1)	0.05	(66 ± 2)	(34 ± 2)	(15 ± 4)	0.07
Subject #1	0.72 ± 0.05	0.37 ± 0.03	0.17 ± 0.03	0.92 ±	0.72 ± 0.04	0.38 ± 0.03	0.17 ± 0.05	0.92 ±
Normal speed	(66 ± 2)	(34 ± 2)	(16 ± 2)	0.06	(66 ± 1)	(34 ± 1)	(16 ± 4)	0.05
Subject #2	0.85 ± 0.09	0.48 ± 0.05	0.16 ± 0.02	0.76 ±	0.80 ± 0.05	0.54 ± 0.07	0.16 ± 0.06	0.75 ±
Slow speed	(64 ± 1)	(36 ± 1)	(12 ± 1)	0.10	(60 ± 2)	(40 ± 2)	(12 ± 5)	0.06
Subject #2	0.65 ± 0.07	0.34 ± 0.06	0.14 ± 0.03	1.04 ±	0.64 ± 0.05	0.35 ± 0.03	0.15 ± 0.05	1.02 ±
Normal speed	(66 ± 3)	(34 ± 3)	(15 ± 4)	0.20	(65 ± 2)	(35 ± 2)	(15 ± 5)	0.08

**Table 4.** Comparison between *νGRF* calculated from the insole and force-platform data: normalized root-mean square error (NRMSE), Pearson correlation coefficient, and standard error in the estimation of the stance phase duration (namely $\text{MAE}(\Delta t_{ST}^{fp}-\Delta t_{ST}^{in})$). Data from slow- and normal-speed walking were grouped together. The last column reports the total number of steps that were both recorded by one of the insoles and the force platform.

	**NRMSE**	**Pearson Correlation**	$\text{MAE}(\Delta t_{ST}^{fp}-\Delta t_{ST}^{in})[\text{s}]$	**# of Recorded Steps**
Subject #1	54.25 ± 9.65	0.88 ± 0.03	0.03 ± 0.03	18
Subject #2	106.09 ± 16.22	0.89 ± 0.03	0.03 ± 0.02	27
